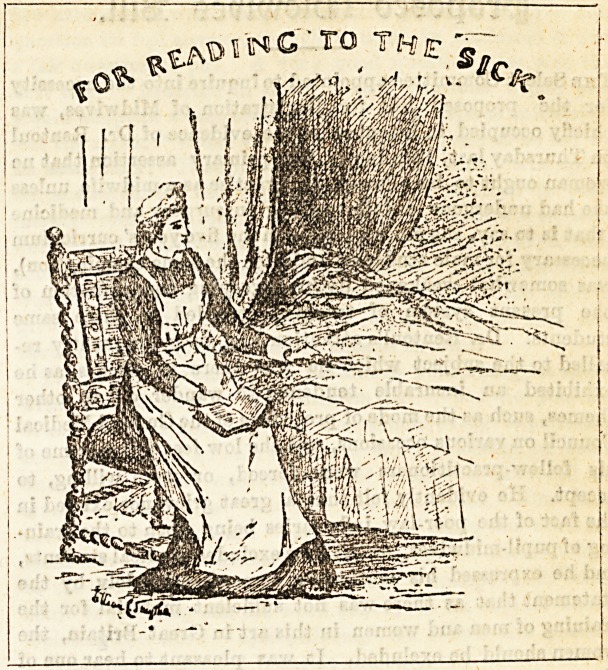# The Hospital Nursing Supplement

**Published:** 1892-05-28

**Authors:** 


### "The Hospital" Nursing Mirror.

Being the Extra Nursing Supplement of "The Hospital" Newspaper.

Contributions for this Supplement should be addressed to the Editor, The Hospital, 140, Strand, London, W.C., and should have the word "Nursing" plainly written in left-hand top corner of the envelope.

### En Passant.

LECTURES TO NURSES.—Professor Klein, F.R.S., on the 14th inst., gave a demonstration with the aid of the oxy-hydrogen light "On the Part Played by Organisms in Disease" to the nursing staff of St. Mary Abbott's Infirmary, Kensington.

THE NURSES' HOSTEL.—There was an "open day" at the Nurses' Hostel, Percy Street, W.C., on the 19th inst., when Miss C. J. Wood invited friends interested in the welfare of nurses to inspect the two houses which she has fitted up for the reception of sojourners in London, either for an occasional night or as a resting place between cases. There are also one or two unfurnished rooms which any nurse wishing to make a kind of permanent home can rent, surrounding herself with such household gods as she may possess. No. 27 has been used for a nurses' hostel for over two years; the second house is a recent addition. Sir Edward Sieveking, Mr. Ernest Hart, Dr. Oswalde Browne, Mr. Warrington Howard, and Dr. Watkins took part in the proceedings, and evinced a kindly interest in the project, and also in Miss Wood's scheme for supplying "the practitioner's daily nurse."

RANDOM EVIDENCE.—In his evidence before the Select Committee, Dr. Rentoul expressed himself as not averse to women doctors, but he thinks there is always friction between midwives and the medical profession; but, being questioned as to possibility of existence of jealousy between the sexes, he sturdily maintained that it would not exist on the part of the doctor, but probably would be all the side of the trained woman In the question of hospital nurses, Dr. Rentoul had also a very strong opinion to offer, to the effect that they were gradually usurping the duties of the students; in fact, women have but a poor character from him, and he speaks of midwives as unmitigated evils, which should be suppressed, and superseded by a race of medical officers to be trained for the last six months of their medical course in this special branch. A reference was made to the doctors who marry midwives or nurses, which did not appear to have any special connec'ion with the proposed Bill; but the oddity of introducing such a subject induced a smiling query as to whether it was the partnership or the marriage which affected the present point.

HOSPITAL REPORTS.—From the point of view of any-one interested in the nursing of a hospital, the hospital report is often entirely void of interest owing to any information on the subject being entirely absent from the very document in which one might reasonably suppose a full, true, and particular account would be found. The number of nurses employed in a hospital might surely be given, and a short interesting statement of the progress of their training knowledge. In one report we have lately received, the Chaplain gives a report, but the Lady Superintendent and the nurses have the veil of silence thrown over their proceedings. Let us hope that next year some of our hospitals will send out reports containing some mention of their nursing staff. It would be a beneficial action on some person's part to alter the dry-as-dust nature of hospital reports generally, for while a bare statement of bare facts may suffice for a regular subscriber who knows the merits of his own particular institution, this does not suffice for the casual reader. It is well to be concise and business-like, but the latter quality need not necessarily exclude every item of interest.

NOTTINGHAM AND NOTTS NURSING ASSOCIATION.—The seventeenth annual meeting of this Association was very successful, and the chair was taken by Mr. R. Evans, J.P. There are two branches of nursing, one of which supplies trained nurses to those who can afford to pay, and the other provides nurses for the sick poor. Much difficulty has been found in increasing the staff of the private nurses, as very few hospitals are willing to train probationers for a private institution. We are glad to see that nurses who have been connected with the Institution for five or nine years are given a bonus.

ANNUAL MEETING OF THE WORKHOUSE INFIRMARY NURSING ASSOCIATION. — At seven o'clock the nurses who had met in the Conduit Street Picture Gallery sat down to tea. Many different uniforms were seen round the table, and several well-known Matrons, amongst others Miss Styring, of Paddington, Miss Wesley, of St. George's-in-the-East, and Miss Hughes, of Kensington, and others. After tea, Lady Wantage gave away medals to many of the nurses, and Miss Twining made a short speech, alluding to the illness of Miss Wilson, who, though at last a little better, was unable to attend, much regret being expressed at her absence. A short concert brought a very pleasant evening to a close.

POOR AND OFTEN FRIENDLESS.—These two terms are often found hand in hand. One of the Lady Superintendents of a London district nursing association says that some of the saddest instances her nurses come across are the cases of poor clerks and governesses, numbers of whom live in cheap lodgings around, and who only have "sufficient for the day," and nothing to fall back upon in case of illness. There never was a better scheme than that of sending out a district nurse. She goes about doing good so quietly and unobtrusively what her help must be to the many lonely folk who fight single-handed in our great City it does not need a great stretch of imagination to conceive.

BLACKPOOL LADIES' SICK POOR ASSOCIATION. —This co-operation is the outcome of the Sick Nursing Association. The town is divided into seventeen different wards, and a lady acts for each ward. The Association provides nourishment for the sick, provided the application when enquired into is found to be made from genuine distress. Also in some special cases when recovery or comfort depend on suitable clothing being forthcoming, the Association sends warm clothing, for, in spite of the undesirability of giving recklessly, there is such a thing as being utterly unable to provide a bit of flannel to wrap round a child recovering from bronchitis, and blankets have a knack of wearing out and becoming poor and useless, and money is, alas! not always forthcoming wherewith to renew them. The deliberate yet kindly working of this Association deserves all praise.

CONCERT AT THE LONDON HOSPITAL. — An excellent concert was given by the students of the London Hospital Medical School on Friday evening, the 20th inst., in their beautiful college library, which was decorated with palms and other plants for the occasion. A large audience, including a goodly show of sisters and nurses, some members of the visiting staff, as well as many friends of the doctors and the hospital workers generally, showed great appreciation of a capital programme. Several changes had to be made for various reasons, which were amusingly explained by Dr. Gilbart Smith, but they certainly in no way affected the success of the entertainment. The violin performance was a real musical treat, and the songs were well chosen, as well as sung, and of the many good concerts which have been arranged by the London students, past and present, this must rank as one of the best.

### Ventilation, Disinfection, and Diet.

By P. Caldwell Smith, M.D.

### VII.—DISINFECTION (*continued*).

Different Pathogenic Forms of Micrococcus — Micrococci Producing Suppuration—Micrococcus of Erysipelas, of Ulcerative Endocarditis—Bacillus Anthracis—Bacillus of Typhoid Fever, of Pneumonia, of Tubercular Disease (Phthisis, &c.), of Leprosy, of Glanders, of Diphtheria, of Tetanus, of Influenza—Spirillum of Asiatic Cholera (Comma Bacillus of Koch)—Spirillum of Relapsing Fever.

Let us now look at the first division, and we shall mention the pathogenic micro-organisms only.

(*a.*) Micrococcus pyogenes aureus.—Golden yellow when grown on potatoes. This is the most common pus-producing organism, and is found in nearly all cases of suppuration, especially in boils, empyema, and all acute abscesses.

(*b.*) Micrococcus pyogenes albus.—Found also in pus, but is white when cultivated. There are also other forms of micrococcus found in pus, the principal one being streptococcus pyogenes, this latter being the more common one in blood poisoning and gangrene.

(*c.*) We now come to an exceedingly important one—the micrococcus of erysipelas—proved to be the cause of this disease. If this micrococcus is inserted into a healthy person it produces the disease in that person in from 15 to 61 hours.

Micrococci have been found in several other diseases in man, although they have not yet been directly proved to be the exciting cause. One of the most important of these is the micrococcus of ulcerative endocarditis, or inflammatory disease of the internal coating of the heart, and also the micrococcus of puerperal fever.

There are also a large number of these germs which produce disease in the lower animals, but which do not do so in human beings.

(2) We now come to the genus Bacillus, to which most of the disease-producing germs belong. (*a*) The first kind of bacillus is a very malignant one, viz., bacillus anthracis, producing in man what is called malignant pustule or wool sorters' disease. This disease is not as common in Scotland as in some parts of England, notably in Yorkshire, where it is got from the hair imported from Russia. (*b*) Bacillus of malignant œdema, not a very common one but occurring in cases of progressive gangrene after severe accidents. (*c*) Bacillus of typhoid fever, found in the bowels, liver, and spleen of patients suffering from that disease. This bacillus is in almost all cases, in my opinion, taken into the system by means of the digestive tract, either by drinking water or milk in which the bacillus is present, or by taking food on which the bacillus has accidently found its way. Another method of infection, I believe a more common one than is generally supposed, is by means of the hands. If a nurse or medical man is dealing with a patient suffering from typhoid, and more especially if the bowel discharges are very frequent, it, I believe, sometimes happens that a few of these bacilli get on to the hands, and unless these are always thoroughly cleaned and disinfected, there is a probability that when the nurse or medical attendant is taking food, the bacillus may be conveyed into the mouth. The spores or seeds of this bacillus exist in large numbers in the excreta, and if not killed by some means or other may remain quiescent for a long time in fields, meadows, &c., if the sewage flows over these, and so be conveyed back by fruit or vegetables to human habitations again. I do not believe that these spores or germs can multiply outside the human body, but a very few of these spores are sufficient to cause a case of typhoid.

(*d.*) Bacillus of pneumonia (or pneumococcus Fraenkel) present in a large number of cases of pneumonia or inflammation of the lungs, more especially when it becomes an epidemic.

(*e.*) Bacillus tuberculosis, the germ of consumption. This germ is the direct cause of all those tubercular diseases which are so fatal at all times of life, and more especially in infancy, childhood, and youth. There are a large number of diseases at one time thought to be different diseases now classed under the term of tubercular. We have phthisis pulmonalis, or consumption of lungs; phthisis abdominalis, or consumption of the bowels; hydrocephalus, or water in the head, more properly tubercular meningitis; lupus, a skin disease attacking the face and cheeks; scrofula; enlargement of glands of neck, &c. You may also have tubercular disease of bones, joints, throat, kidneys, and in all these, without exception, this tubercular bacillus is present.

There are three ways in which this germ may enter the system: (1) By the lungs, which is by far the most common; (2) by the digestive tract; (3) by the skin, as in case of doctors making post-mortems. The germ, in some cases, causes consumption by being swallowed. This is the case in infants taking milk from tubercular cows, and more especially from cows whose udder is affected with tubercular disease. In them the milk is found to be swarming with the germs of tubercular disease, and if taken unboiled will, in some instances, produce tubercular disease of the bowels. In some cases, however, the digestive juices are able to kill the germ, and so prevent it from doing harm. As I have said, however, by far the most common method of infection is by the lungs, and it occurs in this way. The sputum of patients suffering from tubercular disease of the lungs contains a large number of these germs, and if it is not disinfected, or if, as in too many cases among the poorer classes, the expectoration is discharged on the floors, &c., it dries and forms a powdery dust, and is taken up by the air of the room, and in this dust we find the germ of the disease.

(*f.*) Bacillus lepræ, or bacillus of leprosy, found in all cases of true leprosy.

(*g.*) Bacillus mallei, or bacillus of glanders. This disease occurs occasionally in human beings, horses being the principal source of infection.

(*h.*) Bacillus of diphtheria. It was only after great difficulty that this bacillus was demonstrated by Loeffler.

(*i.*) Bacillus of tetanus or lockjaw, found in the soil of certain localities, and if this soil is introduced into a wound, tetanus is likely to follow. The remedy is, of course, to wash all wounds well with a strong antiseptic.

Besides these, bacilli have been found in some other diseases, but we are not yet in a position to say that they are the casual agents in these diseases. There are also a large number of diseases of animals, such as swine erysipelas, or pig typhoid, and fowl cholera, caused by bacilli, but these do not produce disease in man. (*k.*) Bacillus of influenza. This has only recently been discovered, but without doubt it is cause of the disease. (*l.*) A very important germ is the spirillum of Asiatic cholera, or what is usually called the comma bacillus. Koch discovered this in 1884, and since then he has repeatedly confirmed his original experiments. This bacillus is found in large numbers in the stools of cholera patients, hardly ever in the vomited matters, so that, as in typhoid fever, it is the excreta that have to be carefully and thoroughly disinfected. These bacilli do not, as far as is known, form spores; they are, therefore, much more easily killed than the bacilli of some other diseases. Two or three hours' exposure to a dry atmosphere at the ordinary temperature suffices to kill them. Consequently, we see that the old ideas about transport of cholera germs by the air are quite untenable, and that direct infection is the most probable cause. The bacilli will live, however, in moist cultivations for some months, but in sewage, etc., into which they have been introduced, they cannot be detected after twenty-four hours.

(*m.*) Spirillum Obermeyeri, or spirillum of relapsing fever, found in the blood of patients suffering from that disease.

### Prospects of Private Nurses.

The last year has seen a great advance made in the pecuniary position of private nurses, and they have discovered, amongst other things, that the general feeling of the doctors is entirely in favour of their receiving their own fairly-earned fees, whilst that section of the public which can afford the luxury of trained service is much better pleased to write cheques for the individual nurses than for that vague thing, an "institution." It is a kindly, grateful instinct which incites the recipient of much patient care and skill to remunerate the giver of these (in so far as he *can* do it, for money does not clear all debts!) to the best of his ability.

But all great reforms bring abuses in their train, and it seems now as if the moment had arrived for a friendly warning to some of the enterprising nurses who are perhaps too hastily severing their connection with their own training-schools, and endeavouring to start on an independent career. Let them consider thoughtfully this step ere they take it, for if a hospital acts justly towards its trained private nurses, and pays them at starting £40 per annum, rising to £50 or £55; if it provides complete uniform, and also gives them comfortable headquarters between their cases, then they have little to gain, and possibly much to lose, by making a change. When a thoroughly "home-like" provision is made for their brief intervals of rest, and good care is taken of them in sickness, and the rate of annual salaries is such as that already quoted, then indeed nurses ought to be able, with the help of the R.N.P. Fund, or a similar association, to make some provision for the days when work or, at least, active work, should be laid aside. Yet, whilst urging nurses to consider well the seriousness of abandoning any hospital or home where they are honestly treated, we cannot sufficiently condemn those institutions where the payments made to private nurses are not in due proportion to the money earned by their labours, and also where regular holidays are not assured. No trained nurse should be satisfied with less than four weeks annual holiday, and she ought to get, as a matter of right, not of favour, 24 hours' freedom between each of her cases, however light they appear to have been, besides a complete rest for two or three days after each long engagement with serious illnesses, which are more usual experiences after all than what are technically termed "easy cases." The cruelty of sending to a new patient the weary nurse who has just returned from an invalid should be impossible, and we trust it will soon become so, now that the light of publicity has illumined this and other abuses.

It is also difficult to condemn too strongly the lady speculator who starts a private "home" for nurses, to whom she pays low salaries whilst hiring them out for high fees, thereby making a fine income for herself; but this kind of venture is shaken to its very foundations by the newly-aroused spirit of independence; on all sides we hear of higher pay offered, to attract trained labourers.

Whilst asking fair value for services rendered, exorbitant demands should be avoided, for these are times of increasing competition in the women's labour market; also, it must be remembered, that days of sickness will come when no fees are earned, and some weeks will be "slack" when there is no demand. The nurse, "on her own account," is so in every sense of the word, for no one shares the expenses necessarily incurred at bad seasons any more than they share the earnings of good times.

### Appointment.

Princess Alice Memorial Hospital.—Miss Margaret Cameron, late Assistant Superintendent at Westminster Hospital and Home for Nurses, has been elected by the Committee of Management to the post of Matron at this hospital. There were 48 applications.

### "HUMAN LOVE."

We have been thinking lately of heavenly love and of the happiness which follows when the feeling is mutual between God and man. Love, however, seldom goes upwards with the same strength that is descends—hence we find that God's love infinitely exceeds anything we do or can offer to Him, just as an earthly parent loves its child with a tenderness and depth of affection which is seldom, if ever, returned in its entirety. The higher love is purely unselfish, the lower thinks of itself and its own interests; its gratitude is for favours to come, not for those already received. We see in the animal world, too, the ceaseless care of the mother for her offspring, which the young ones repay by trying at the first opportunity to free themselves from her control. And young people act in a similar manner, and like to find their amusements outside the home circle, voting it "slow" to be contented with the happiness which comes to their own hearth.

How more than foolish is it for us men and women to spurn God's love thus, and get away from the restraints of religion. He the wise Householder rules His household with love and wisdom, His only care is for the well-being of all under His charge. Should not His children love, honour, and obey such a Master?

How our hearts would burn and glow within us if we could realize all that Christ has done for us in the past, all He is doing for us now, all the joys He has prepared for us in the future. The first step towards it is to place ourselves at the feet of Jesus Christ and offer up our hearts to Him, and then strive to make the most of the time of "merciful visitation" which He gives us in illness, and to think of all the kindnesses which He has shown to us. Were not the whole thirty-three years He spent on earth, spent in doing good to the sad and suffering? Did He not bear the punishment which we deserved, and by dying for us, save every child of man from getting his due? How can we be cold and lifeless when we think of all this? But the question is, "Do we ever think of it?" No, we cannot, or we should not be such loveless beings. Let us try to keep this model of perfection before our eyes, and ponder on and imitate the love which He bore to all about Him. Can we not, with the beloved disciple, lean on his breast and draw fresh draughts of love from the intimacy, to be returned in faithful service, among His people? We want to love Him with an unselfish love, too, simply because He loved us, and loves us to the end. Let this, then, be our aim, it will lighten our troubles, soothe our anguish, and make easy our sick bed, if the burden of our song is the goodness of God.

So would I love Thee, dearest Lord,
And in Thy praise would sing,
Solely because Thou art my God,
And my most loving King.

### Proposed Midwives Bill.

THE Select Committee appointed to inquire into the necessity for the proposed Bill for Registration of Midwives, was chiefly occupied in listening to the evidence of Dr. Rentoul on Thursday last, 19th inst. His primary assertion that no woman ought to be allowed to practise as a midwife unless she had undergone a full training in surgery and medicine (that is to say, she must go through the five years' curriculum necessary for male students, and pass the same examination), was somewhat weakened by his sweeping condemnation of the present system of teaching accorded to these same students. Dr. Rentoul's attention had to be frequently re-called to the subject which he was there to discuss, as he exhibited an incurable tendency to wander off to other themes, such as the mode of procedure of the General Medical Council on various occasions, and the low fees which some of his fellow-practitioners were forced, or were willing, to accept. He evidently felt that a great grievance existed in the fact of the poor-law infirmaries being open to the training of pupil-midwives, whilst they excluded medical students, and he expressed his sentiments somewhat crudely by the statement that as there was not sufficient material for the training of men and women in this art in Great Britain, the women should be excluded. It was pleasant to hear one of the gentlemen on the Committee suggest humanely that it might be fairer to say, there were too many doctors in existence, instead of complaining that there were too few patients.

"Competition" is evidently Dr. Rentoul's bugbear; he rings out the word with inconceivable scorn—he asserts again and again that only economy induces women of any class to retain the services of one of their own sex in preference to the one of which he is the representative. How far his medical brethren may choose to be represented by himself is open to question after his statements to the Select Committee become public property! His accusation of forged certificates, an expression which he insisted on reiterating in spite of the suggestion made by more than one gentleman that it was not only a serious, but an unsupported statement, and his sweeping condemnation of all statistics which did not agree with his own, as "inaccurate," all these and many more went to fill up the measure of Dr. Rentoul's discontent with the existing, and, to confirm his somewhat illogical objection, to any alteration in the present order of things. It was gently suggested for his consideration that some of the abuses of which he spoke would disappear before the protection offered to women by the proposed Bill, but he simply remarked that "everyone" would call herself a midwife then, just as, under the Dental Act, barbers and chemists had registered themselves as dentists. A free discussion ensued, and Dr. Rentoul wound up his remarks by an assertion that certificates of proficiency would have no value as they could be easily forged!—a suggestion of fraud which was heard with but little patience. Horrible details of malpractice committed to the knowledge of himself or his friends were wisely cut short by the Chairman, who evidently thought that such disclosures in such a place would tend either to the benefit of decency or morality, being, moreover, mere hearsay. It was most satisfactory to note the calm deliberation and justice with which the various subjects brought forward were considered, though it must have required more than ordinary patience to sift the grain from the chaff in such a bulk of extraneous matter as was brought forward.

We hope to see the statistics which were given on Dr. Matthew Duncan's authority receive some further corroboration before such startling figures receive credence. It was small wonder that Dr. Farquharson and others required their repetition and careful preservation, for who would otherwise dare to think he heard correctly such facts as the following, that of first labours, viz., of all women confined with a first child, one in 15 died! Also that in spite of increased knowledge of ventilation, antiseptics, &c., the practice of midwifery had undergone a retrograde movement in the last ten years!

It was somewhat cheering to find, in spite of other drawbacks, that women's training as midwives includes rather more lectures and attendance on quite as many cases as the medical student—also that patients, and especially country people, far prefer being attended by one of their own sex who performs for them also many of the duties of a thoroughly trained nurse. Mrs. Malleson's evidence was exceedingly interesting, being drawn from her own personal experience during many years of intimate acquaintance with a country district of eight square miles. When she first obtained the service of a trained parish nurse she did so with the local doctor's concurrence on condition that the person employed "should not be a midwife." This was some years ago, and at present she has a midwife installed who is doing most satisfactory work, with the full consent of the medical man; it being arranged that the former should not take any case to which the doctor had been previously called in. Mrs. Malleson spoke of the terrible fatalities which had taken place when "the woman who goes out," and who is absolutely untrained is in sole charge of the poor woman in her hour of need.

Dr. Drage, who was the next speaker, had collected a mass of evidence—which could be only glanced at on account of the lateness of the hour, and is therefore held over till the next meeting of the Committee. He went into the matter of medical education—on similar lines to Dr. Rentoul—but his accusation that the present movement was an attempt to provide partly-trained (and therefore incompetent) persons for the poor, who are attracted by the lower fees (we suppose), is not altogether in harmony with the suggestion of Dr. Rentoul, who said the better he was paid the better his work. We may remember gratefully the clever and good men who give their services, the best of their skill, and their scientific knowledge, freely and lavishly to those too poor to pay their fees—the fees of a consultant, not the half-crown of which our Liverpool medico spoke with such scathing contempt. Dr. Drage referred with some disgust to the suggestion that a properly trained midwife would assuredly judge correctly of the proper moment at which, in the case of abnormal or delayed labour, the aid of the doctor should be secured; he remarked that this was an all important matter of which the doctor himself could alone judge. Perhaps, at the next sitting of the Committee he will kindly explain how this is to be managed. It is a question whether most general practitioners could spare the time to watch for themselves—they usually, we believe, have to leave this point, to a certain extent, to the discretion of the woman friend, be she nurse or near relative, who is generally forthcoming at the time of need. Perhaps during those "years and years" when this young practitioner says he was "waiting for" cases of midwifery to "enlarge the experience he had gained in hospital," he might have been willing to do this patient "waiting until called for," but even then his assertion that the doctor is the only person who can summon himself without fear of error, might not be accurately acted up to. The meeting closed with a very general feeling that Dr. Rentoul had unwittingly proved himself to be a most valuable witness as to the absolute need for an immediate and trustworthy register for properly trained midwives.

### Wants and Workers.

*The Lady Superintendent*, Home for Incurables, Upper Parliament Street, Liverpool, writes on behalf of one of the patients, who would be most grateful for the artificial arm, if suitable, and not already disposed of.

*Miss Linnell*, 17, Elm Park Gardens, Chelsea, S.W., would be glad to hear of a piano to be given away. It can be sent to Foulis Ward, Brompton Hospital.

### For Asylum Attendants.

*(From a Correspondent.)*

THE nursing examination questions of the Medico-Psychological Association have been a surprise in some respects. One would like very much to know whether the gentlemen who set the papers ever read the handbook for attendants, published under the auspices of the association, and recommended by the association as a basis for study and examination. It is admitted without question that the faculty of examining well, is as much a born faculty as the poetic, and while examiners have to make up their minds for perverseness and stupidity in answering questions, candidates have likewise the privilege of giving a *quid pro quo* and criticising the perverseness and stupidity of some of the questions put before them. If there is one thing more certain than another in the scheme of asylum training, it is this, that teachers and examiners unconsciously teach and examine over the heads of their pupils. It takes some little time to get down to the level of education and intelligence of attendants and nurses, and this is not said with any feeling of invidious distinction, but rather because we feel from experience that the two cannot see eye to eye unless they understand each other better. It is natural for the teacher, especially if he be a medical assistant, to aim at neatly turned sentences and well rounded periods, but the business in hand is teaching and examining more than the mere practice of public speaking, though we do not see why the two should not be combined with success, for the simplest language is after all the most eloquent.

To return to the questions of the recent examinations; we note two in particular that seemed to stump the candidates. Those are the fcurth and the tenth. An asylum attendant or nurse is expected to know a good deal, but to expect them to define the word "everish" is too much in the present early state of this new scheme of training, and because they are not nurses of ordinary hospitals where simple or malignant fever is a prevalent symptom. They are asked, "What observations would you make in a case where you thought the patient was feverish?" and examiners, as well as candidates, might well puzzle over the meaning of this question. They may mean anything and everything, and it is impossible to fix a standard answer with standard value, even in the mind of the man who set the question himself. Of course, this is more or less true of a great many questions, and a rigid line of demarcation is impossible in many answers, especially in questions of this kind. Yet all the same it has to be remembered that the average asylum nurse's mind is not elastic, is not trained to adapt itself to different points of view, and is incapable of *finesse* or making bold ventures in the answer of dubious questions.

The tenth question, "What precautions would you adopt in a case of paralysis, and what events would you look out for?" has been no less a trial and worry to many candidates. In the first place the word "paralysis" is one of wide meaning, and includes a variety of types that neither a hospital nor an asylum nurse could possibly be expected to explain or understand. How then are they to answer the question, "What precaution would you adopt in a case of paralysis?" or to say, "What events would you look out for?" The candidates naturally understood "paralysis" to mean general paralysis, and from the asylum point of view it was a very good thing to do, as that is the form of paralysis most common in asylums, and calling most for a nurse's care and forethought.

The twelfth question, "What is the insane ear?" has a certain sarcasm in it as applied to attendants' examinations, as it is well known that attendants and nurses are too readily blamed for this deformity. There was, therefore, a *naïvété* in the candidate's reply, an unconscious simplicity in the answer, "due to a blow," or "a knock," but there are few attendants who really were in a position to give this question its full answer, though it must be confessed it was a fair question to put. A very good question also was the first one, "Describe in detail the precautions which you would adopt in a case committed to your care as suicidal;" but the sixth, "What varieties of cases require to be fed by an attendant? Describe the procedure in each," was apt to be answered on the supposition that it referred to cases merely refusing food. Several candidates, however, were quite equal to answering it very fully, better, perhaps, than some of their examiners could have done. This particularly was the case with sick-ward attendants, who were able to give a remarkable variety of cases requiring to be fed, and each having individual peculiarities of its own.

The seventh question, "A patient in your charge is seized with an epileptic or epileptiform fit. What do you do? What do you observe?" is one which every experienced attendant should be able to answer well, provided he really understood what was required of him, and provided the word "epileptiform" was left out altogether. Now there are many fits which are "epileptiform," using the word in a loose way, and as medical men are not agreed themselves as to the precise significance of the word "epileptiform," what sense or meaning can the word convey to an asylum nurse? Deleting the word "epileptiform," we follow the question "What do you do?" "What do you observe?" Now here is a question requiring a certain elasticity of mind to understand what the full portent of the answer should be. The night attendant will only think of epileptic fits at night. The day attendant will be guided in framing his answer by his experience in the ward with it may be the patient placed in a single room off the day-room, where epileptics may sleep off their seizures. The dining-hall attendant will be most impressed with the symptoms and dangers of epileptic seizures occurring during meals, but the combined experience of the three is necessary to give the full answer to the question. Yet there is still one more difficulty. Some candidates describe fully what they do, and what they observed during the fit, and there their answer ends. Would the examiners regard that as a full answer to this question? or would they also require a statement of what is done, and what is observed in the post epileptic state?

All these criticisms, notwithstanding this examination paper, to those who fully comprehend the draft of the questions was a searching one, and eminently practical. There can be no doubt that questions of this kind will give to attendants and nurses in asylums a fresh impetus in the study and discharge of their work among the insane.

MEDICO-PSYCHOLOGICAL ASSOCIATION NURSING EXAMINATION.

We have been favoured with the following results from the undernoted asylums:—

CRICHTON ROYAL INSTITUTION, DUMFRIES. — Isabella Gribeson, 164½; Mary Johnstone, 156; Maggie Clarke, 154; Joseph Ormston, 151; Jemima Riddock, 147; Edith Thorburn, 146; Jessie McKay, 146; Elsie Anne Leslie, 145; Barbara Scott, 144; Robert Cooper, 140; Jane Williamson, 138; Marian Cocker, 136; Mary Dolan, 134; John Campbell, 129; Alice Noble, 126; Peter McArthur, 121.

STIRLING DISTRICT ASYLUM, LARBERT.—Kate Dunbar, 164½; James Sim, 157; M. Malfeather, 149; John Lawson, 149; William Fraser, 147.

GLASGOW DISTRICT ASYLUM, BOTHWELL. — Donald McMillan, 165½; John Campbell, 150½; Isabella Henderson, 146½; Hugh McEwan, 134.

The total possible number of marks was 180.

We shall be glad to receive intimation of results from other asylums.

### Everybody's Opinion.

*[Correspondence on all subjects is invited, but we cannot in any way be responsible for the opinions expressed by our correspondents. No communications can be entertained if the name and address of the correspondent is not given, or unless one side of the paper only be written on.]*

THE DANGER OF DEVELOPING INTO A TYRANT.

"Vera" writes: It is a strange, sad, but undeniable fact that if a woman is placed in a position of power she is apt to degenerate into a tyrant. And this fact, alas, is the rule, not the exception, as I have been forced to admit, after an experience of a variety of women in various positions of authority. This unfortunate failing of our sex is perhaps more marked in hospitals than elsewhere, because there women are placed in almost unbounded authority over their subordinates for the time, and it has been a matter of interest to me to watch and wonder over the especially marked effect upon hospital sisters of the absolute power they enjoy. Here, for example, we have a woman who, as probationer, is pleasant, charming, and courteous to her fellow workers, and popular amongst nurses of every grade. "Ah! she is nice enough *now*; wait till she is a sister," are the ominous words we hear applied to her by some cynical on-looker. And the words are too often amply justified, the change from the probationer's to the sister's dress seeming to bring with it a change of character as well. Of course there are exceptions, and some women seem capable of wielding the sceptre and still retaining their loveableness and courtesy. My own hospital training was throughout, a singularly happy one, but I have often longed to have this motto illuminated for almost every sister in the place: "Manners are not idle, but the fruit of loyal nature and of noble mind." I frankly own that probationers can be irritating beyond words at times, but is it not probable that we should do far more with our pros., and train them equally well, if we were invariably gentle, and never brusque with them? Perhaps sisters too soon forget the time when they themselves were learners. If they would only remember how much easier it is to work for a person who has a pleasant word in passing, and who, when possible, bestows upon one a word of praise, how different some wards would be! It costs so little to be gentle and courteous and patient, and it makes just the whole difference to the people who are working with you, and it has often made me almost afraid, to find how easy it is to gain the love of the nurses and ward-maids under one's sway. A perfect nurse must not only be patient with the sick folk under her care, but she must feel quite as much bound to learn patience towards these fellow women, who are often tired, whose nerves get unstrung, and tempers irritated as much as do our own. A nurse very often needs as much patient, helpful care from the sisters as the patients themselves. It is false reasoning to say that there must be a hard and fast line drawn between sisters and nurses, or authority cannot be kept up. That is the purest, most unmitigated nonsense. Authority there needs must be, but sisters and nurses alike are doing the same work, and authority is only strengthened, never weakened, when rulers and ruled work as friends. "Be courteous," said the great apostle. There is nothing weak, or idle, or small in being good mannered. Manners show the man, for to all time good manners will be the fruit of "loyal nature and of noble mind."

### Hints to Nurses.

We have received a tin of Moser's Pure Soluble Cocoa, and have found it very excellent. A teaspoonful mixed with boiling water makes a breakfast cupful of cocoa, which is quite free from the disagreeable greasy taste so often found in preparations of this kind. A cupful of this cocoa would be a very good thing for anybody sitting up at night, as it is easy to prepare, and is undoubtedly sustaining.

Flett's Fragrant Skin Cream.—One of the best skin creams we have met with hails from Scotland; it is neither sticky nor greasy, and a few drops of it rubbed well into the skin certainly cures roughness or redness in a very satisfactory manner. It has a delicious scent, and would be invaluable in a nursery, its composition being guaranteed absolutely harmless to the tenderest skin. It is moderate in price, and may be had from Mr. A. Flett, Murray Place, Uddingston, N.B.

### Where to Go.

Many institutions, besides private householders, are at a loss to provide suitable entertainments, either to add to their funds, or to amuse their friends (as the case may be), at a reasonable expenditure of time and money, the former being frequently as important a matter as the latter. The professional entertainers who can take the whole amusement of an evening into their own hands are very few, and consequently they become perhaps too well known, and variety is demanded. Mr. Heathcote, himself one of these rare individuals, has realised the weakness of the position, and is, we think, in supplementing his own successful efforts, supplying a decided want. He has formed a small drawing-room dramatic troupe, who can be engaged at a moderate cost, for the performance of popular plays, many of his own productions being especially suitable for the purpose. To introduce his scheme to the public he gave a dress rehearsal, so to speak, of nine items of his repertoire at the Albert Hall, a short time since, which were enthusiastically received by large audiences. Mr. Heathcote has been fortunate in securing the assistance of most able artists, and we feel sure that his troupe will give great satisfaction to those who obtain their services. Many of the pieces in the programme are for two performers only, which places an excellent performance within the reach of most would-be entertainers.

### Nurses' Residential Club.

A large and friendly party assembled on 23rd inst., at 92, Charlotte Street, Fitzroy Square, which has been recently opened as a Nurses' Residential Club. The house has been cleverly adapted to its present requirements, and the little cubicles are capitally suited for those birds of passage—private nurses. The sitting-room is cheerful and bright, and so is the dining-room, and an excellent bath is available for the modest charge of twopence, whilst the meals are also moderate in cost and well served. An arrangement made with the Secretary enables non-resident nurses to share in the obvious benefit of having a permanent address for their letters.

### Notes and Queries.

### Answers.

J. C. C.—We could not use your contribution. We agree entirely with your sentiments, but not with your rhyme. Why not write your opinion in "Everybody's Opinion."

*A Holiday in Switzerland.*—We have heard from "Nurse in Yorkshire," and have received her references, and have forwarded all letters. The scheme must be properly worked out, and a definite idea formed of the sum which the holiday will cost each member of the expedition.

*Station Gate, Skipton, Bristol, Bosworth, and others.*—All letters have been forwarded to "Nurse in Yorkshire."

*Matron.*—We will insert your news with much pleasure.

*Hospital Reader.*—The "Hospital Annual" will give you information as to the number of beds, staff, &c., of the hospitals, obtainable at this office. If you write stating what particular ones you wish to know about we will answer you. We regret that only urgent replies can be given a written answer.

*Z.*—The Workhouse Infirmary Nursing Association, 6, Adam Street, Adelphi, W.C., trains free in midwifery, but nurses are bound for three years after training, and must already be trained nurses.

*An Aged Nurse.*—Why have you given us no name and no address? We can do nothing for you till we know where you live and who you are. Write to "Nursing," at this office.

*G. M., Cornwall.*—We cannot find room for the whole of your letter, as our columns are very full just now.

*N. C., Bath.*—Am writing to Bath for you, and will let you know result in these columns as soon as possible.

*M. C. L., Dundee.*—St. George's Hospital, Hyde Park Corner, would take you, perhaps, if there is a vacancy. Then there is the Mater Misericordia, in Dublin, to which you could apply. The founders of hospitals have a perfect right to make what rules they consider best for the general well-being of their institutions. You must remember that many institutions of your own faith would not admit a Protestant. Tolerance, however, happily grows apace.

*Dictionary.*—"Nurses' Dictionary," 140, Strand, post free, 2s. "Hoblyn's Dictionary of Medical Terms," Whittaker and Co., Ave Maria Lane.

*Nurse Isabel.*—Write to Matrons of King's College Hospital, the London, and the Royal Free, for their rules.

## Figures and Tables

**Figure f1:**